# Tau and TDP-43 accumulation of the basal nucleus of Meynert in individuals with cerebral lobar infarcts or hemorrhage

**DOI:** 10.1186/s40478-019-0700-z

**Published:** 2019-03-28

**Authors:** Hiroyuki Hatsuta, Masaki Takao, Akane Nogami, Akiko Uchino, Hiroyuki Sumikura, Tadayuki Takata, Satoru Morimoto, Kazutomi Kanemaru, Tadashi Adachi, Tomio Arai, Masato Hasegawa, Shigeo Murayama

**Affiliations:** 1 Department of Neuropathology, Tokyo Metropolitan Institute of Gerontology, 35-2 Sakae-cho, Itabashi-ku, Tokyo, 173-0015 Japan; 2 Department of Neurology, Tokyo Metropolitan Geriatric Hospital, 35-2 Sakae-cho, Itabashi-ku, Tokyo, 173-0015 Japan; 3 Department of Pathology, Tokyo Metropolitan Geriatric Hospital, 35-2 Sakae-cho, Itabashi-ku, Tokyo, 173-0015 Japan; 4 Department of Neurology, Osaka City University, Graduate School of Medicine, 1-4-3 Asahi-machi, Abeno-ku, Osaka, 545-8585 Japan; 5 Hatsuta Neurology Clinic, 11-3-205, Kuzuha-hanazono-cho, Hirakata-shi, Osaka, 573-1121 Japan; 6 Department of Neurology, Saitama Medical University International Medical Center, 1397-1, Yamane, Hidaka-shi, Saitama, 350-1298 Japan; 7 Department of Neurology, Tottori University, 86 Nishi-cho, Yonago-shi, Tottori, 683-8504 Japan; 8 Dementia Research Project, Tokyo Metropolitan Institute of Medical Science, 2-1-6 Kamikitazawa, Setagaya-ku, Tokyo, 156-8506 Japan

**Keywords:** Basal nucleus of Meynert (BNM), Cerebral hemorrhage, Cerebral infarction, Cerebrovascular disease, Neurofibrillary tangles (NFTs), Tau, TAR DNA-binding protein 43 (TDP43)

## Abstract

A previous study reported that a massive cerebral infarct in the territory of the middle cerebral artery (MCA) may be associated with development of neurofibrillary tangles (NFTs) in the ipsilateral basal nucleus of Meynert (BNM). We analyzed 19 cases of an MCA territory infarct and 12 with a putaminal hemorrhage (mean age 82.5 years; female/male ratio 8/23; mean time from stroke onset to autopsy 4182 days). In both groups, 74–100% had a significantly higher rate of phosphorylated tau immunoreactive or Gallyas Braak silver stain-positive neurons on the BNM-affected side than on the BNM-unaffected side. These NFTs were immunoreactive for anti-RD3 and anti-RD4 antibodies, and a triple-band pattern was observed by immunoblot analysis with anti-tau antibody. Most NFTs might be formed within the 5–10 years after stroke onset. There were significantly more TAR DNA-binding protein 43 (TDP43) immunoreactive structures on the BNM-affected side than on the BNM-unaffected side. We showed that many NFTs with TDP43-immunoreactive structures were observed in the ipsilateral BNM associated with a massive cerebral infarct in the MCA territory or a putaminal hemorrhage.

## Introduction

Tau is a protein that binds to and stabilizes microtubules, which is required for maintaining neuronal shape and transport of cellular cargo [3]. Hyperphosphorylated tau is the major component of helical or straight filaments in degenerating neurons and/or glial cells in many neurodegenerative diseases. Abnormal tau accumulation is usually observed in the somata of neurons and glial cells. These filamentous tau aggregates contribute to pathologies of the central nervous system, such as Alzheimer’s disease (AD) and other tauopathies.

A previous study reported that massive cerebral infarct in the territory of the middle cerebral artery (MCA) on one side of the hemisphere is associated with the development of numerous neurofibrillary tangles (NFTs) in the basal nucleus of Meynert (BNM) on the side ipsilateral to the infarct [11]. Those results, however, have not been well analyzed by other researchers or for other types of stroke.

In addition, as described in detail previously TAR DNA-binding protein 43 (TDP-43)-immunoreactive deposition can be detected in neurodegenerative tauopathy, including Alzheimer disease [2, 16], Pick disease [4], corticobasal degeneration [19], progressive supranuclear palsy [20], and argyrophilic grain disease [5]. In addition, aggregation of TDP-43 may affect cognitive impairment in deing points using tissue samples registered in the Brain Bank for Aging Research (BBAR; http://www.mci.gr.jp/BrainBank/). 1) We wanted to clarify whether NFTs are present in BNMs in individuals with an ischemic stroke as well as those with a hemorrhagic stroke. 2) If NFTs are present, are there TDP-43 deposits in the BNM?

## Materials and methods

### Cases

Tissue samples were obtained from autopsy materials that were collected at the Tokyo Metropolitan Geriatric Hospital and the Institute of Gerontology [1, 6, 10]. Among them, we analyzed all cases of cerebrovascular disease (CVD) (including cerebral infarcts or hemorrhage) with a lobar lesion. For this study, we defined a lobar infarct or hemorrhage as involving more than one-third of the MCA area. We excluded cases with brain tumors or neuroinflammatory diseases.

Three neuropathologists reviewed each case separately and then conferred to reach the final diagnosis. Clinical information was obtained retrospectively from medical charts and summaries and was reviewed by two board-certified neurologists.

### Neuropathologic analysis

#### Sampling and routine and immunohistochemical staining

Brains and spinal cords were examined according to our BBAR protocol [1, 6, 10]. The brains and spinal cords were fixed in 20% buffered formalin (Wako, Osaka, Japan) for 7–13 days and then dehydrated in a graded alcohol series, cleared in xylene, and embedded in paraffin. Serial sections (6 μm thick) were cut and stained with hematoxylin and eosin and by the Klüver–Barrera method. They were further examined with Gallyas Braak silver staining [7].

For immunohistochemistry, we used the Ventana Benchmark XT autoimmunostainer (Ventana, Tucson, AZ, USA) according to the manufacture’s protocol [1, 6, 10]. The BNM sections were immunostained using the following antibodies raised against synthetic peptide corresponding to phosphorylated tau (ptau; AT-8, 1:100, monoclonal; Innogenetics, Ghent, Belgium), phosphorylated α-synuclein (pSyn#64, polyclonal, 1:20,000; Wako, Osaka, Japan), and phosphorylated TDP-43 (pTDP43; pSer409/410, monoclonal, 1:10,000; Cosmo Bio, Tokyo, Japan). The signals from monoclonal and polyclonal antibodies were detected using the automatic system on a Ventana Discovery with the I-View DAB Universal Kit (Roche, Basel, Switzerland) according to the manufacturer’s instructions. Sections were counterstained with hematoxylin.

#### Analysis of the BNM

The cerebrum was sliced in coronal sections vertical to the anterior and posterior commissure line. The BNM sections [12] at the level of the anterior commissure line were immunostained using the following antibodies raised against synthetic peptide corresponding to AT8: pSyn#64, pSer409/410, anti-3-repeat tau (RD3, monoclonal, 1:2000; Merck, Darmstadt, Germany), and 4-repeat tau (RD4, monoclonal, 1:50; Merck) and (anti-4R, monoclonal, 1:500; Cosmobio). They were further examined with Gallyas Braak silver staining [7].

#### Quantitative analysis of phosphorylated tau immunoreactive (ptau+) neurons in the BNM

To obtain the number of ptau+ neurons, we counted only the number of the neurons with nucleoli. The immunoreactive density of total BNM neurons was calculated. In addition, the ratio of RD3−/RD4- or anti-4R immunoreactive neurons was calculated.

#### Semi-quantitative analysis of pTDP43 immunoreactive (pTDP43+) neurons in the BNM

We semi-quantitatively analyzed the immunohistochemical staining with pTDP43 antibody. Our grading system was modified based on the scoring system of a previous study [18]: Neuronal cytoplasmic inclusions (NCIs), glial cytoplasmic inclusions (GCIs), and neuronal intranuclear inclusions (NIIs) immunoreactive for pTDP43 were quantitatively analyzed and scored on a scale of 0–3, depending on the total number of pTDP-43+ NCIs, GCIs, or NIIs: 0 = none; 1 = 1–3; 2 = 4–9; 3 = ≥10. pTDP43+ dystrophic neurites (DNs) were semi-quantitatively scored as 0–3, where 0, absent; 1, sparse; 2, moderate; 3, severe.

### Immunoblot analysis

The sarkosyl-insoluble fractions were prepared as described by Taniguchi-Watanabe et al. [17]. Frozen BNM (0.25 g) from one MCA territory infarct case (case 1) was homogenized in 20 volumes (5 ml) of buffer A (10 mM Tris-HCl, pH 7.5, containing 1 mM EGTA, 10% sucrose, and 0.8 M NaCl). After addition of Sarkosyl (final concentration at 2%), the homogenate was incubated for 30 min at 37 °C and spun at 20,000×g for 10 min at 25 °C. The supernatant was removed, transferred to 1.5-mL tubes,+ and ultracentrifuged at 100,000×g for 20 min at 25 °C. The pellets were washed by ultracentrifugation with 0.5 mL of sterile saline, solubilized in sodium dodecyl sulfate-sample buffer, and subjected to 4–20% gradient polyacrylamide gel (Wako) for electrophoresis. Transferred proteins on PVDF membrane was probed with the antibodies to tau T46 (Thermo) at 1:1000, RD3 at 1:500, RD4 at 1:500 and anti-4R at 1:1000, biotinylated 2nd antibody, avidin–biotin complex (Vector) and developed with diaminobenzidine and nickel chloride.

### Statistical analysis

The Mann-Whitney’s U test was used to analyze differences in NFTs or pTDP43+ structures between the BNM-affected and BNM-unaffected sides. The Spearman’s rank correlation coefficient was used to analyze correlation between the numbers of anti-RD3 antibody-immunoreactive (RD3+) and anti-RD4 antibody-immunoreactive (RD4+) or anti-4R immunoreactive (4R+) neurons. Statistical analysis was performed using SPSS 15.0J software (SPSS, Chicago, IL, USA). Statistical significance was set at *p* < 0.05.

## Results

### Clinical and pathologic studies

Among the 23 lobar infarct cases (female/male 6/17, mean age 82.7 [SD 7.4] years), infarcts were found in the anterior (ACA), middle (MCA), and posterior (PCA) cerebral artery territories in 1, 19, and 3 cases, respectively (Table 1). Among the 17 lobar hemorrhage cases (female/male = 5/12, mean age 81.6 [SD 9.6] years), 12 were putaminal hemorrhage. Other hemorrhage sites were a frontal lobe (*n* = 1), occipital lobes (*n* = 2), cerebellum (n = 1), and brain stem (n = 1) (Table 1). The mean interval from the onset of stroke to death (autopsy) in the MCA territory infarct cases was 3500 (SD 2721) days, and that for the putaminal hemorrhage was 5262 (SD 4223) days.

**Table 1:** Profile of lobar infarct and lobar hemorrhage cases

		Infarct cases					Hemorrhage cases				
case	side	Braak stage	interval (days)	age (y.o.)	sex	case	side	Braak stage	interval (days)	age (y.o.)	sex
	*MCA territory*						*putamen*				
1	R	1	177	74	M	24	R	1	967	82	M
2	L	1	606	74	M	25	R	1	2720	58	M
3	R	1	1787	84	M	26	L	1	5017	81	M
4	L	1	2790	74	M	27	R	1	5955	83	M
5	L	1	3511	75	M	28	L	1	10,415	89	M
6	R	1	3975	85	M	29	R	2	2922	65	M
7	R	1	4821	71	M	30	R	2	5844	95	F
8	R	1	5114	92	M	31	R	3	1592	84	M
9	L	1	5114	77	M	32	L	3	2526	88	M
10	L	1	9869	87	M	33	L	4	4383	81	F
11	L	2	119	74	M	34	L	5	16,048	92	F
12	R	2	1707	83	M	35	R	6	4749	85	F
13	R	2	3525	82	F						
14	R	2	7305	90	M		*Frontal lobe*				
15	L	2	7345	79	M	36	L	5	2376	82	F
16	R	3	4502	82	M						
17	L	4	344	102	F		*Occipital lobe*				
18	L	4	730	91	F	37	L	1	7305	79	M
19	L	5	3168	86	F	38	L	2	3288	88	M
	*ACA territory*										
20	R	2	3891	75	M		*Cerebellum*				
	*PCA territory*					39	L	2	1820	86	M
21	L	1	1144	81	M						
22	R	1	4564	87	F		*Brain stem*				
23	L	2	5516	83	F	40	L	1	529	69	M

*Braak stage* Braak neurofibrillary stage, *interval* interval from the onset of stroke to death (autopsy), *MCA* middle cerebral artery, *ACA* anterior cerebral artery, *PCA* posterior cerebral artery, *R* right, *L* left, *F* female, *M* male

### NFTs of the BNM in CVD cases

**Fig. 1:**
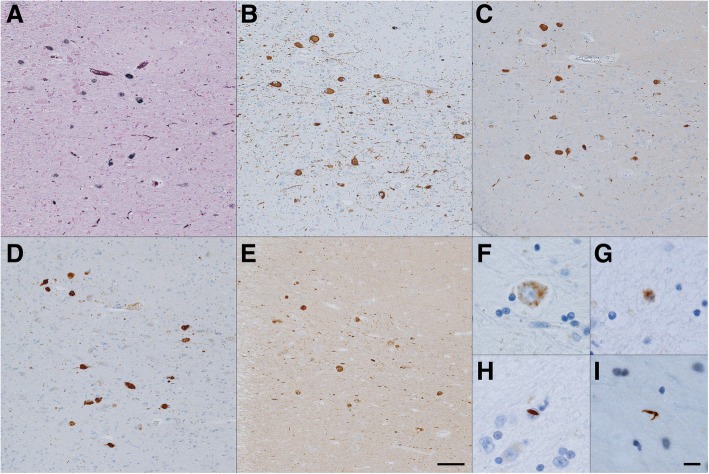
Gallyas Braak stain in the BNM (**a**). Immunocytochemistry by anti-tau antibodies AT8 (**b**), RD3 (**c**), RD4 (**d**) and anti-4R (**e**) in the BNM. Immunocytochemistry by anti-phosphorylated TDP-43 antibody in the BNM. (**f**) Granular neuronal cytoplasmic inclusion (NCI). (**g**) Granular glial cytoplasmic inclusion (GCI). (**h**) Cat’s-eye neuronal intranuclear inclusion (NII). **(i)** Thread-like structures (dystrophic neurites, DNs). Bar = 100 μm (**a-e**), 10 μm (**f-i**)

**Fig. 2:**
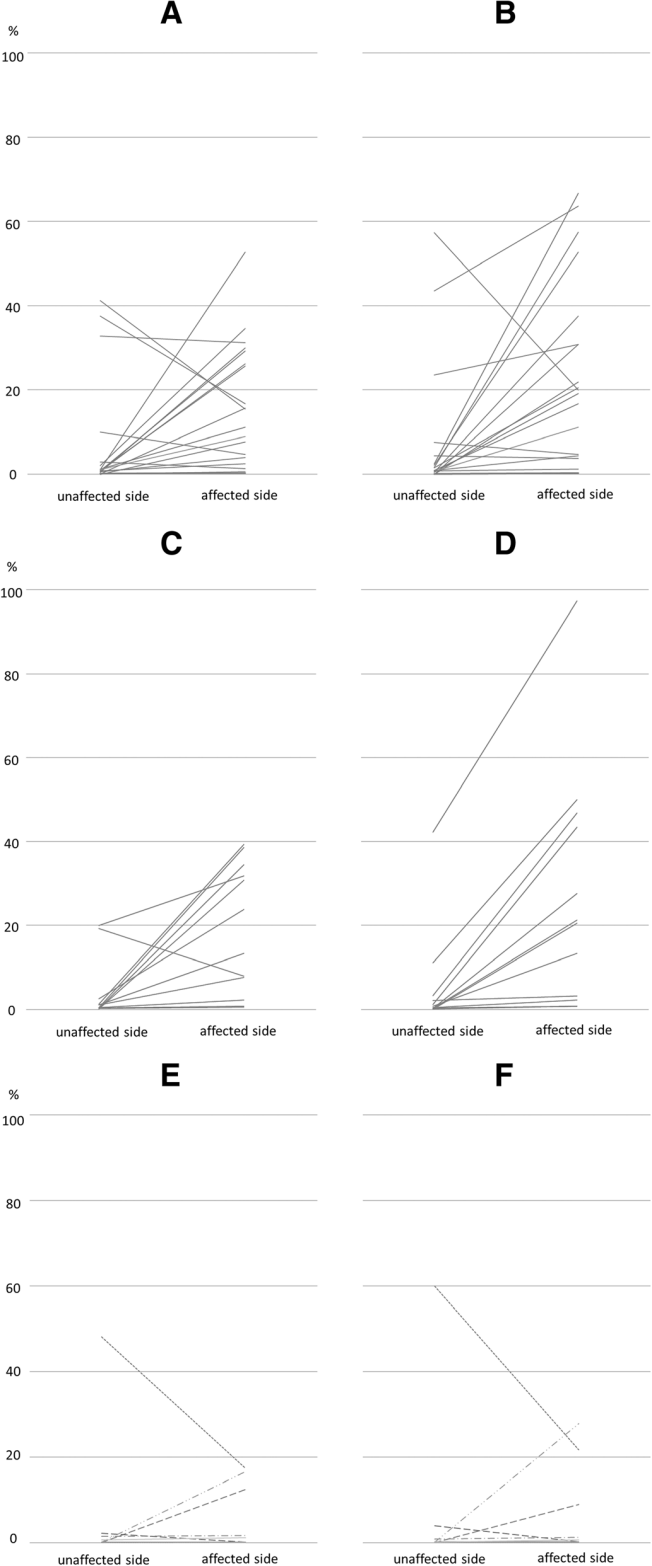
Rate of neurofibrillary tangles (NFTs) in neurons of the basal nucleus of Meynert (BNM) of cardiovascular disease (CVD). (**a**, **b**) The 19 middle cerebral artery (MCA) territory infarct cases. (**c**, **d**) The 11 putaminal hemorrhage cases. (**e**, **f**) Other CVD cases. (**a**, **c**, **e**) Phosphorylated tau-immunoreactive (ptau+) neurons. (**b**, **d**, **f**) Gallyas Braak stain-positive (GB+) neurons. (**a**) In most cases (14/19, 74%), the ratio of ptau+ neurons in total neurons was higher on the BNM-affected side than on the unaffected side. The median rate was also significantly higher on the BNM-affected side than on the unaffected side (*p* < 0.01). (**b**) In most cases (16/19, 84%), the rate of GB+ neurons was higher on the BNM-affected side than on the unaffected side. The median rate was also significantly higher on the BNM-affected side than on the unaffected side (*p* < 0.01). (**c**) In most cases (11/12, 92%), the rate of ptau+ neurons was higher on the BNM-affected side than on the unaffected side. The median rate was significantly higher on the BNM-affected side than on the unaffected side (*p* < 0.01). (**d**) In all 11 cases (100%), the rate of GB+ neurons was higher on the BNM-affected side than on the unaffected side. The median the rate was significantly higher on the BNM-affected side than on the unaffected side (*p* < 0.01). (**e**, **f**) In the other CVD cases, there were no significant differences in ptau+ or GB+ BNM neurons on the affected and unaffected sides

In most MCA territory infarct cases (14/19, 74%), the rate of ptau+ neurons was higher on the BNM-affected side than on the BNM-unaffected side (Figs. 1b, 2a). The median rate was significantly higher on the BNM-affected side than on the BNM-unaffected side (*p* < 0.01).

- MCA territory infarct cases (16/19, 84%): The rate of Gallyas Braak stain-positive (GB+) neurons was higher on the BNM-affected side than on the BNM-unaffected side (Figs. 1a, 2b). The median rate was significantly higher on the BNM-affected side than on the BNM-unaffected side (*p* < 0.01).
- Putaminal hemorrhage cases (11/12, 92%): The rate of ptau+ neurons was higher on the BNM-affected side than on the BNM-unaffected side (Fig. 2c). The median rate was significantly higher on the BNM-affected side than on the BNM-unaffected side (*p* < 0.01).
- Putaminal hemorrhage cases (11/11, 100%): The rate of GB+ neurons was higher on the BNM-affected side than on the BNM-unaffected side (Fig. 2d). The median rate was significantly higher on the BNM-affected side than on the BNM-unaffected side (*p* < 0.01).
- Other CVD cases: There were no significant differences in the number of ptau+ or GB+ BNM neurons between the BNM-affected and BNM-unaffected sides (Fig. 2e, f).

Furthermore, because of the similar results in each stroke subtype, we also studied 19 MCA territory infarct cases and 12 putaminal hemorrhage cases together (mean age 82.5 years; female/male = 8/23; mean time from the stroke onset to autopsy 4182 days).

### Braak NFT stage and NFTs of the BNM in MCA territory infarct and Putaminal hemorrhage cases

**Fig. 3:**
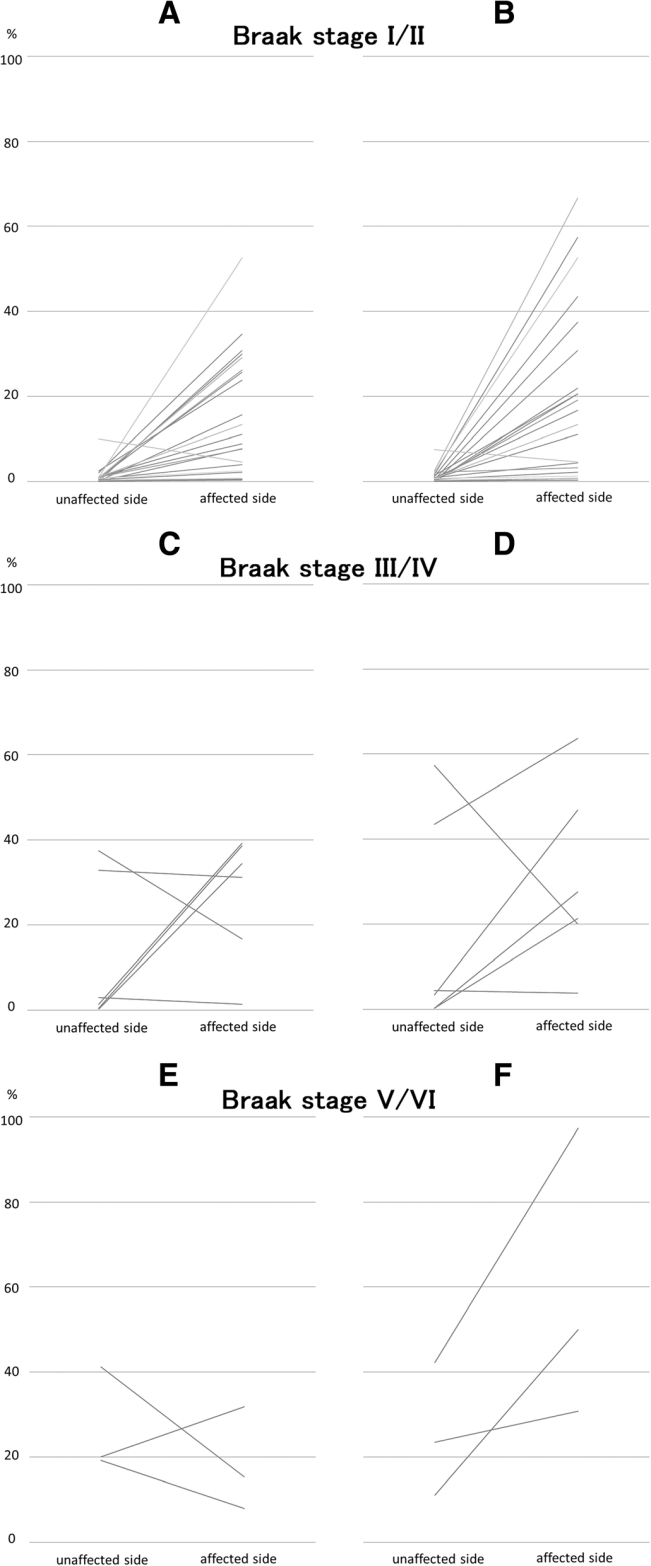
Rate of NFTs in neurons of the BNM cases of infarcts in the MCA territory and putaminal hemorrhages, by Braak NFT stage. (**a**, **b**) Braak NFT stages I and II cases. (**c**, **d**) Stages III and IV cases. (**e**, **f**) Stages V and VI cases. (**a**, **c**, **e**) Phosphorylated tau-immunoreactive (ptau+) neurons. (**b**, **d**, **f**) Gallyas Braak stain-positive (GB+) neurons. (**a**) In most cases (21/22, 95%), the rate of ptau+ neurons was higher on the BNM-affected side than on the unaffected side. The median rate was significantly higher on the BNM-affected side than on the unaffected side (*p* < 0.01). (**b**) In most Braak NFT stage I and II cases (21/22, 95%), the rate of GB+ neurons was higher on the BNM-affected side than on the unaffected side. The median rate was significantly higher on the BNM-affected side than on the unaffected side (*p* < 0.01). (**c-f**) No differences in ptau+ or GB+ BNM neurons on BNM-affected and unaffected sides

In most Braak NFT stage I and II cases (22/31, 71%), the rate of ptau+ neurons was higher on the BNM-affected side than on the BNM-unaffected side (21/22, 95%), (Fig. 3a). The median rate was significantly higher on the BNM-affected side than on the BNM-unaffected side (*p* < 0.01). In addition, in most Braak NFT stage I and II cases, the rate of GB+ neurons was higher on the BNM-affected side than on the BNM-unaffected side (21/22, 95%) (Fig. 3b). The median rate was significantly higher on the BNM-affected side than on the BNM-unaffected side (*p* < 0.01). In Braak NFT stage III and IV (6/31, 19%) or V and VI (3/31, 10%) cases, there were no differences in the ptau+ or GB+ neurons between the BNM-affected and BNM-unaffected sides (Fig. 3c–f).

### Tau isoform analysis of NFTs in the BNM

**Fig. 4:**
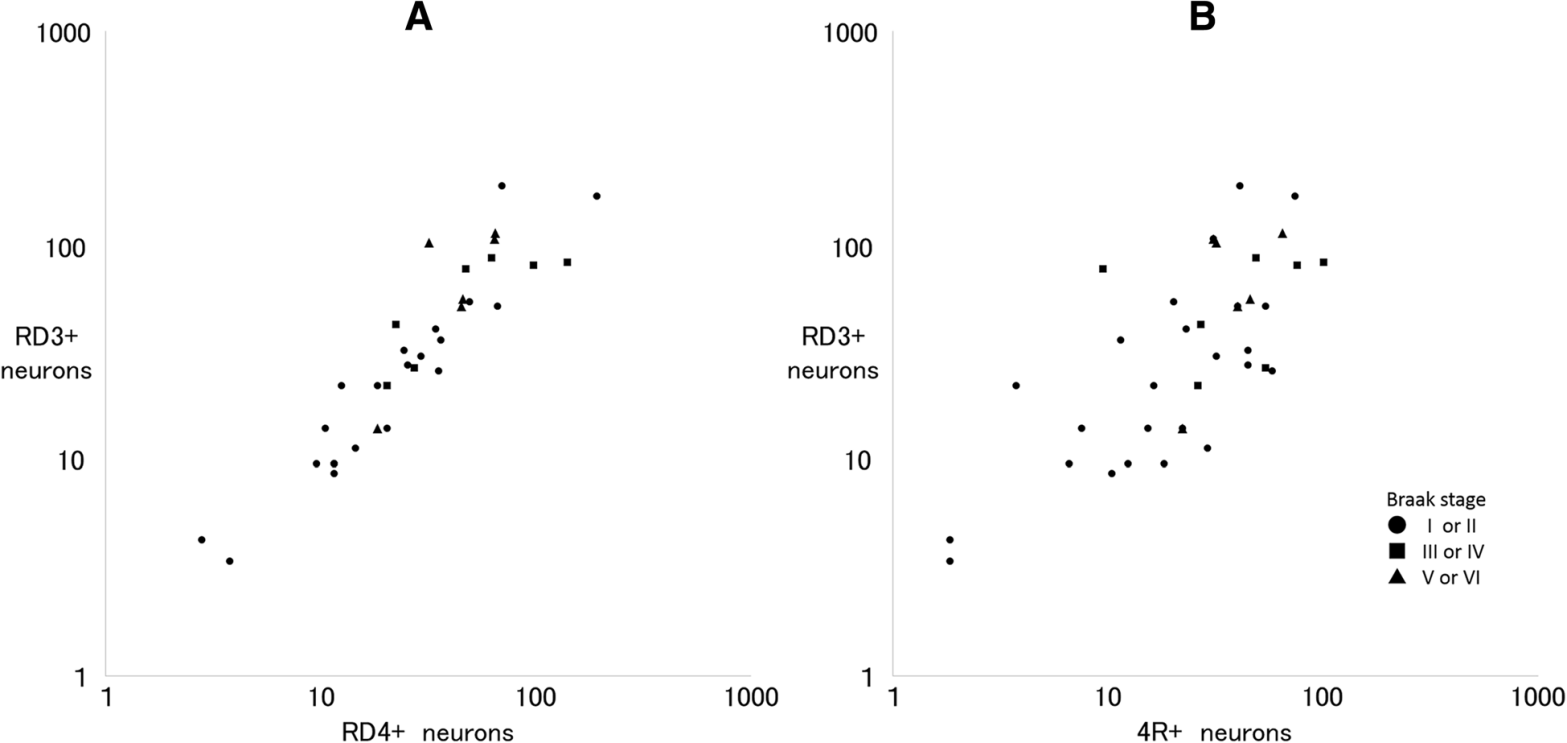
RD3+ and RD4+ (**a**) or anti-4R+ (**b**) neurons of the BNM in cases of MCA territory infarcts and putaminal hemorrhages. There were more anti-RD3 antibody immunoreactive (RD3+) neurons when there were more anti-RD4 antibody immunoreactive (RD4+) neurons, regardless of Braak stage. Except for stage V or VI (r = 0.26, *p* = 0.83), the numbers of RD3+ and RD4+ neurons were strongly correlated (total: r = 0.78, *p* < 0.01; stage I or II: r = 0.83, *p* < 0.01; stage III or IV: r = 0.82, *p* = 0.046) (**a**). The total number of RD3+ and 4R+ neurons or in stage I or II were strongly correlated (total: r = 0.78, *p* < 0.01; stage I or II: r = 0.69, *p* < 0.01), whereas there was no correlation in stage III or IV (r = − 0.14, *p* = 0.78) or stage V or VI (r = − 1.00) (**b**)

RD3+, RD4+ and 4R+ neurons were observed in the BNM of the 31 cases (Fig. 1c-e). The number of RD3+ neurons increased simultaneously with the RD4+ or 4R+ neurons regardless of the Braak stage (Fig. 4). Thus, except for the neurons in Braak stage V or VI (r = 0.26, *p* = 0.83), the number of RD3+ and RD4+ neurons were strongly correlated (total: r = 0.78, *p* < 0.01; stage I or II: r = 0.83, *p* < 0.01; stage III or IV: r = 0.82, *p* = 0.046) (Fig. 4a). The total number of RD3+ and 4R+ neurons or in Braak stage I or II were strongly correlated (total: r = 0.78, *p* < 0.01; stage I or II: r = 0.69, *p* < 0.01), whereas there was no correlation in stage III or IV (r = − 0.14, *p* = 0.78) or stage V or VI (r = − 1.00) (Fig. 4b).

### NFTs of the BNM and the interval from stroke to death

**Fig. 5:**
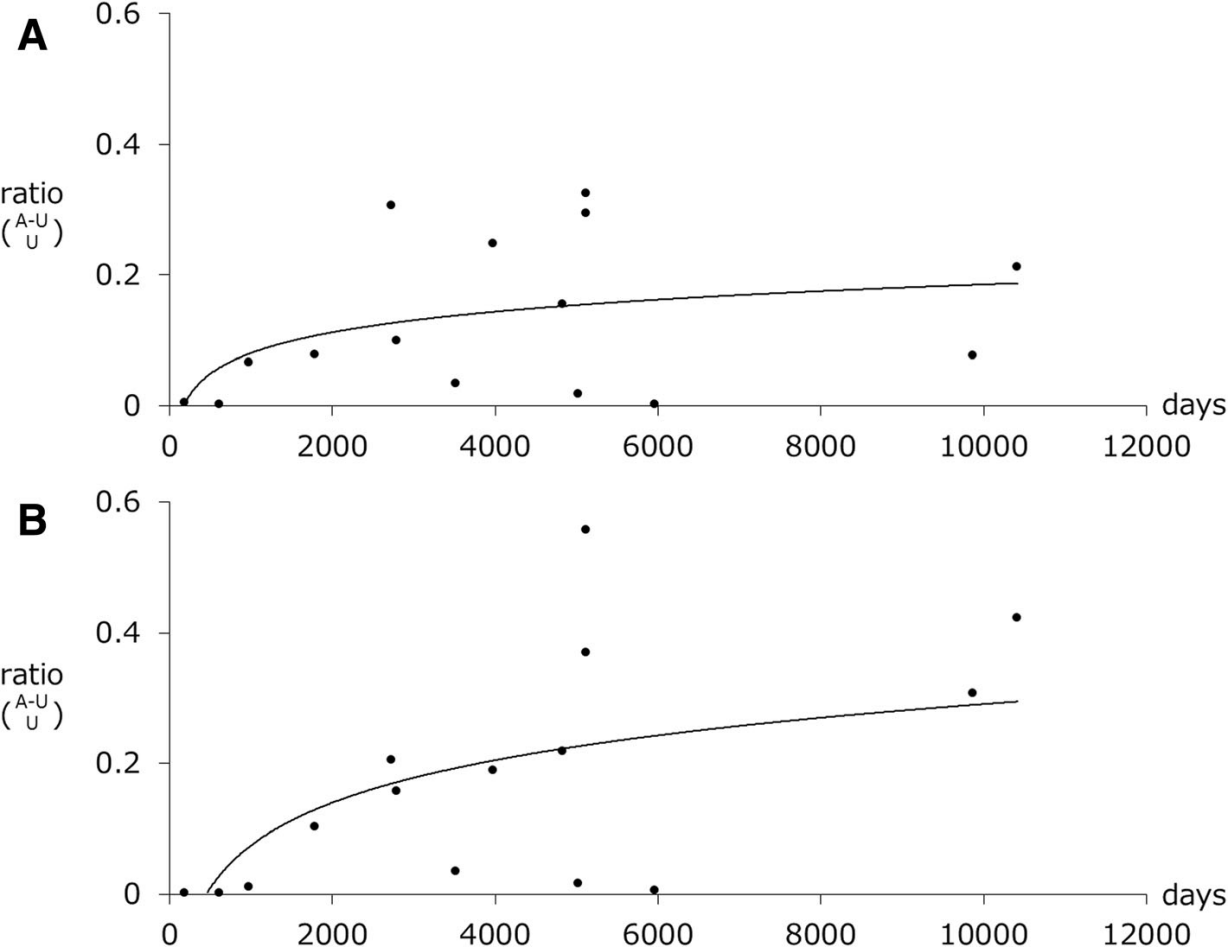
NFTs in the BNM and the time interval from stroke to death. (**a**) In the 22 Braak stage I and II cases, the rate of the differences in the number of phosphorylated tau-immunoreactive (ptau+) neurons between BNM-affected and BNM-unaffected sides and was correlated with the time interval from stroke to death (r = 0.48, *p* = 0.02). (**b**) The rates of the number of GB+ neurons and the stroke–death interval were correlated (r = 0.69, *p* < 0.01). According to the logarithmic curves, NFTs increased during the 5–10 years following stroke onset until death

We compared the difference in the NFT numbers between BNM-affected and BNM-unaffected sides and the time interval from stroke to death (Fig. 5). In the 22 Braak stage I and II cases, the difference in ptau+ or GB+ neurons in both numbers and stroke–death intervals were strongly correlated (r = 0.48, *p* = 0.02; r = 0.69, *p* < 0.01, respectively). The logarithmic curves with the best fit to a series of data points are shown in Fig. 5. The trajectories of the curves indicate that the number NFTs continued to increase for 5–10 years after stroke onset (until the patient died).

### pTDP43+ structures in the BNM

**Fig. 6:**
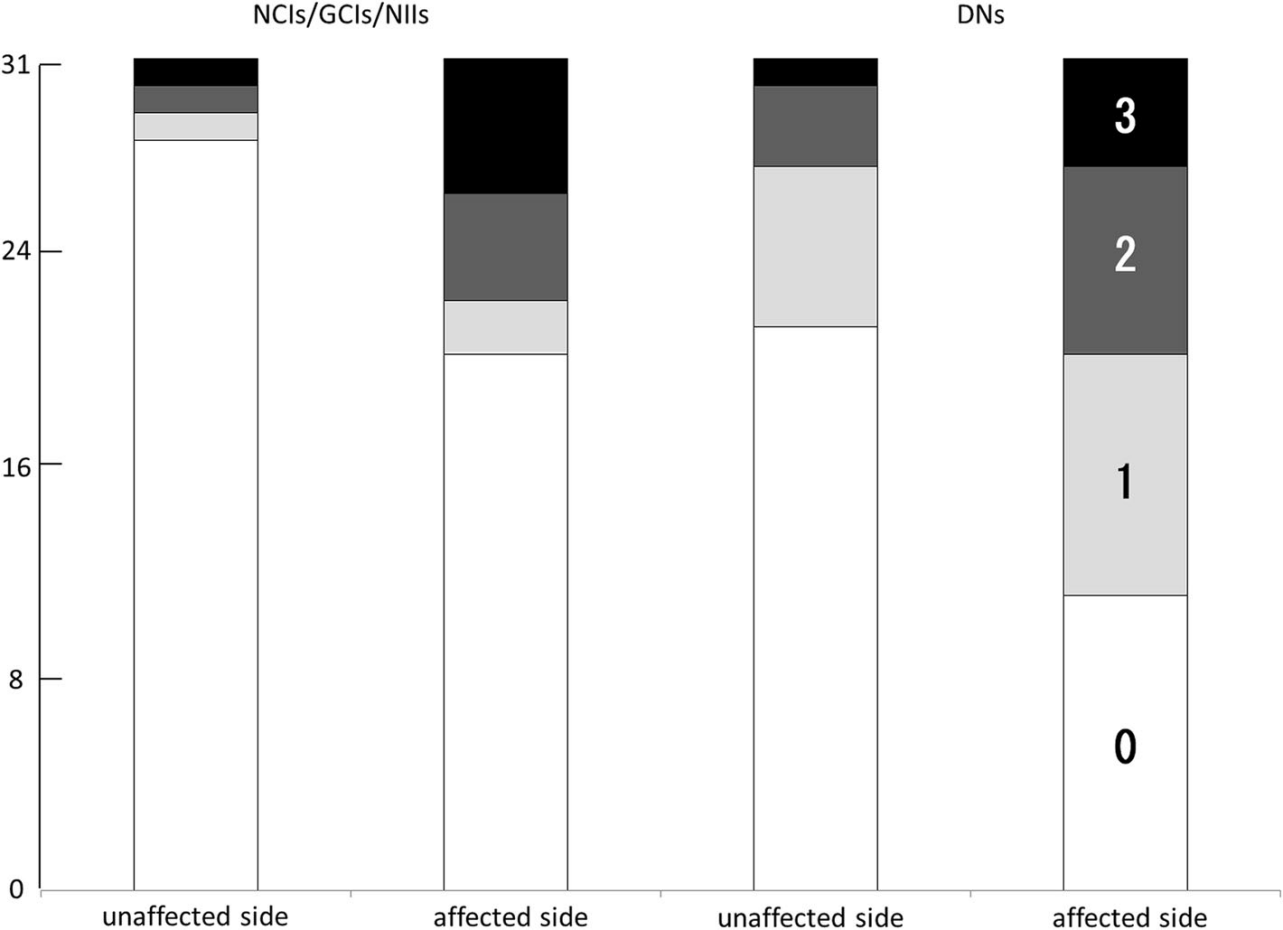
Grading the anti-phosphorylated TDP-43 (pTDP-43) structure in the BNM. pTDP-43 immunoreactive neuronal cytoplasmic inclusions (NCIs), glial cytoplasmic inclusions (GCIs), and neuronal intranuclear inclusions (NIIs) were semi-quantitatively scored as 0–3 depending on their total number: 0 = none; 1 = 1–3; 2 = 4–9; 3 = ≥10. pTDP-43 immunoreactive dystrophic neurites (DNs) were semi-quantitatively scored as 0 to 3: 0, absent; 1, sparse; 2, moderate; 3, severe. The grades of the pTDP immunoreactive NCIs/GCIs/NIIs and DNs were significantly higher on the BNM-affected side than on the BNM-unaffected side (*p* = 0.01, *p* < 0.01, respectively)

Various pTDP43+ structures—i.e., granular NCIs and GCIs, thread-like structures (DNs), dot-like structures of neuropil—were observed in the BNM (Fig. 1). pTDP43+ cat’s-eye NIIs were rare (Fig. 1). pTDP+ NCIs/GCIs/NIIs and DNs grades were significantly higher on the BNM-affected side than on the BNM-unaffected side (*p* = 0.01, *p* < 0.01, respectively) (Fig. 6).

### Immunoblot analysis

**Fig. 7:**
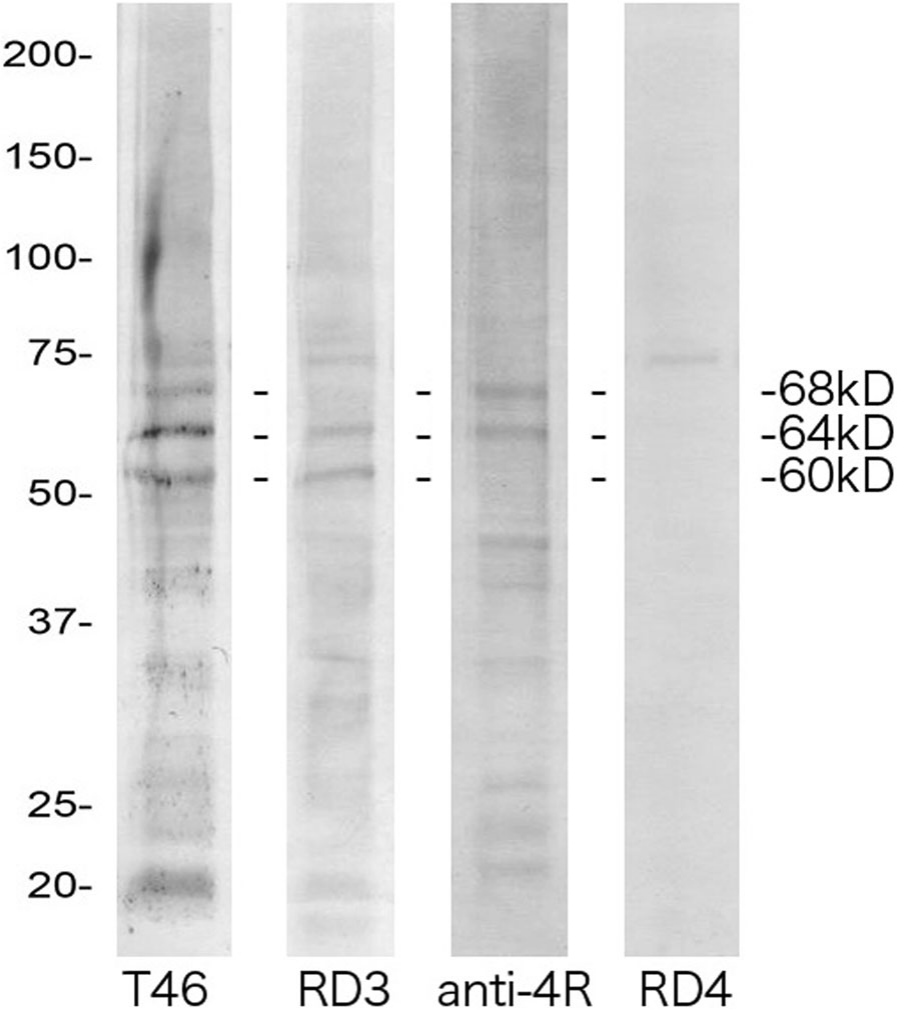
Immunoblotting of sarkosyl-insoluble fractions with anti-tau antibodies T46, RD3, anti-4R, and RD4. Frozen BNM tissue from one case of an MCA territory infarct was subjected to immunoblotting. Three major abnormal tau bands of 60, 64, and 68 kDa were detected with T46. The lower 60- and 64-kDa bands were detected with RD3, and the upper 64- and 68-kDa bands were detected with anti-4R. RD4 failed to detect these bands. The pattern of these tau bands was indistinguishable from that seen in Alzheimer’s disease

Figure 7 shows the results of immunoblotting of sarkosyl-insoluble fractions from case 1 (with an MCA territory infarct and anti-T46, RD3, anti-4R, and RD4 antibodies). Three major abnormal tau bands of 60, 64, and 68 kDa were detected. The pattern of these tau bands was indistinguishable from those seen in AD.

## Discussion

We showed that 1) there were many NFTs in the ipsilateral BNM of a case of massive cerebral infarct in the MCA territory or putaminal hemorrhage on one side; 2) most NFTs might be formed during the 5–10 years after stroke onset and before death; and 3) pTDP43+ structures were observed with NFTs in cases of massive CVD.

### Massive CVD and tau

A previous study reported that there were numerous NFTs in the BNM in cases of a massive MCA-area infarct that had paired helical filaments, making them AD-type NFTs [11]. In our study, immunohistochemistry showed that these NFTs were positive for anti-RD3, anti-RD4, and anti-4R antibodies. In addition, immunoblot analysis with anti-tau antibody revealed a triple-band pattern. These immunobiochemical analysis were consistent with AD pattern.

We believed that the putaminal hemorrhage as well as massive MCA-area infarcts may cause numerous NFTs in the BNM on the ipsilateral side. In addition, the results did not depend on the Braak stage. Asymmetrical neuronal shrinkage, between affected and unaffected sides, without actual changes in neuronal numbers, may result in apparent differences in neuronal numbers. However, it might be assumed that the BNM-affected side neurons ipsilateral to the infarct would be more likely to shrink after an infarct than the BNM-unaffected side neurons, and that this shrinkage would tend to reduce, rather than increase, the BNM-affected side neuron counts. In most those cases, number of NFTs in the BNM may reach the maximum number the 5–10 years after stroke onset, with the interval from stroke to death being a good fit on logarithmic curves. As this must be inferred from cross-section data (at death), this result can only be tentative.

As described in detail previously [13], chronic traumatic encephalopathy (CTE) is a type of progressive neurodegenerative disease caused by repetitive mild traumatic brain injury, characterized by widespread deposition of hyperphosphorylated tau, which appears as NFTs [14]. As tau accumulation in the BNM associated with CVD as well as CTE tauopathy have in common that tau accumulates after 5–10 years or more due to destruction, both may be caused by the same mechanism.

### Massive CVD and TDP-43 lesions

Diseases with secondary TDP-43 pathology include AD and hippocampal sclerosis [2, 16], Guam parkinsonism–dementia complex [8], Pick disease [4], corticobasal degeneration [19], progressive supranuclear palsy [20], argyrophilic grain disease [5], and Lewy body disease [15]. Widespread TDP-43-immunoreactive inclusions have been observed in more than 80% of CTE cases [17]. pTDP-43-immunoreactive structures and NFTs are often observed in the BNM of massive CVD cases. There are multiple other biological mechanisms by which head injury may trigger the molecular pathways leading to neuronal degeneration in CVD, including inflammation, glutamate excitotoxicity, and oxidative stress.

Tau accumulation in neurons in the BNM might result in a gradual cognitive decline of unknown cause in patients who had a first large stroke but lacked a recurrence. Further, considering that neurons in the BNM project their axons to widespread areas of the ipsilateral cerebral cortex including the frontal cortex [9], together with the ability of tau aggregates to propagate along neuronal pathways, it is also possible that the NFT formations in the BNM subsequently cause abnormal tau accumulation in remote and wider regions, including the frontal cortex. The BNM is rich in acetylcholine and choline acetyltransferase, and one of pharmacological treatments of cognitive decline such cases having one large CVD may focus on compensating for a faltering BNM function through artificially increasing acetylcholine levels.

Our study was limited by the small number of cases. To our knowledge, however, no other analyses of bilateral BNM samples of patients with cerebral hemorrhage in autopsy materials have been reported. In addition, we present the relation between NFT formation and the period during which NFTs are formed, from the vascular event or pTDP-43 accumulation to death (stroke onset–death interval).

## Conclusion

We presented cases of massive cerebral infarct in the territory of the MCA or putaminal hemorrhage on one side of the brain. We focused on the NFTs with pTDP-43-immunoreactive structures in the BNM-affected side (with the infarct) and the ipsilateral non-affected side. Our findings may contribute to revealing the mechanism of NFT formation and/or the development of BNM-targeted therapy.
